# The metabolome of [2-^14^C](−)-epicatechin in humans: implications for the assessment of efficacy, safety, and mechanisms of action of polyphenolic bioactives

**DOI:** 10.1038/srep29034

**Published:** 2016-07-01

**Authors:** Javier I. Ottaviani, Gina Borges, Tony Y. Momma, Jeremy P. E. Spencer, Carl L. Keen, Alan Crozier, Hagen Schroeter

**Affiliations:** 1 Mars, Inc., McLean, VA 22101, USA; 2 Department of Nutrition, University of California Davis, Davis, CA 95616, USA; 3 Department of Food and Nutritional Sciences, University of Reading, Whiteknights, Reading RG6 6AP, UK

## Abstract

Diet is a major life style factor affecting human health, thus emphasizing the need for evidence-based dietary guidelines for primary disease prevention. While current recommendations promote intake of fruit and vegetables, we have limited understanding of plant-derived bioactive food constituents other than those representing the small number of essential nutrients and minerals. This limited understanding can be attributed to some extent to a lack of fundamental data describing the absorption, distribution, metabolism and excretion (ADME) of bioactive compounds. Consequently, we selected the flavanol (−)-epicatechin (EC) as an example of a widely studied bioactive food constituent and investigated the ADME of [2-^14^C](−)-epicatechin (300 μCi, 60 mg) in humans (n = 8). We demonstrated that 82 ± 5% of ingested EC was absorbed. We also established pharmacokinetic profiles and identified and quantified >20 different metabolites. The gut microbiome proved to be a key driver of EC metabolism. Furthermore, we noted striking species-dependent differences in the metabolism of EC, an insight with significant consequences for investigating the mechanisms of action underlying the beneficial effects of EC. These differences need to be considered when assessing the safety of EC intake in humans. We also identified a potential biomarker for the objective assessment of EC intake that could help to strengthen epidemiological investigations.

The crucial role of primary prevention in public health is undisputed. The role of diet and nutrition in the promotion and maintenance of good health throughout all stages of life cannot be overestimated, and the need for the development of evidence-based dietary guidelines is well recognized. Current recommendations strongly promote the intake of fruits and vegetables^1^. However, although fruit and vegetables contain a vast number of bioactive constituents, our current understanding of their impact on human health is limited to a relatively small number of nutrients and minerals established to be essential for human life (e.g. vitamin C) and those very few non-essential nutrients, hereafter referred to as bioactives, for which a dietary guideline or daily reference intake has been established (e.g. dietary fiber)^2^. Consequently, there is an increasing need to comprehensively understand the potential health benefits of bioactives. The demand for evidence-based insights and information about the health benefits of bioactives does not just come from the scientific community, but also from the public at large, as evidenced by the plethora of daily reports in the media on the putative benefits of a wide variety of foods ranging from green tea, blueberries, cocoa, and olive oil to red wine.

A critical assessment of the outcomes of current research in the wider area of bioactives and human health highlights significant gaps^3^ that can be attributed, to some extent, to a general lack of data describing the absorption, distribution, metabolism and excretion (ADME) of these compounds in humans. The availability and greater appreciation of ADME data will impact on all aspects of research in this area, including 1) the development of objective biomarkers of intake, and thus the interpretation of epidemiological data on associations between intake and health; 2) the assessment of safety and risks associated with intake; 3) the design and execution of dietary intervention studies and, perhaps most importantly, 4) the use of cell cultures *in vitro* and organ-/tissue preparations *ex vivo* that are aimed at elucidating the mechanisms of action that causally underlie observations *in vivo*. The latter point is of particular interest in relation to polyphenol and flavonoid research, as polyphenolic plant constituents such as (−)-epicatechin, (−)-epigallocatechin-3-*O*-gallate, quercetin, resveratrol and hesperetin comprise a large group of dietary compounds, many of which are currently being investigated in the context of health and nutrition^3^,^4^,^5^.

Regrettably, most of the hundreds of papers published in the context of investigating potential mechanisms of action of polyphenols using cell culture systems do not take into consideration the ADME of these compounds, generating data sets with limited utility. As almost all polyphenolic bioactives are subject to extensive metabolism *in vivo*, there is a clear need for testing their metabolites in *ex vivo* and *in vitro* model systems rather than the native compounds that occur in foods or plant extracts. Considering the above, and aiming ultimately at supporting an evidence-based approach to the development of dietary recommendations and applications, we studied the ADME in humans of a commonly consumed dietary flavonoid, namely the flavanol (−)-epicatechin (EC). The rationale for using EC as a representative example for bioactives, for which comprehensive data on ADME are needed, is as follows:

1. Present in tea, pome fruit, cocoa, wine and various berries, EC is one of the most widely consumed flavanols, and it is absorbed and present in the systemic circulation of humans^5^,^6^.
2. A considerable number of dietary intervention studies investigating the intake of flavanols such as EC have established their potential role in the primary and secondary prevention of cardiovascular disease and the secondary prevention of diabetes^7^,^8^,^9^,^10^,^11^.
3. EC is one of the few nutrients for which a causal relationship between intake and the modulation of cardiovascular function has been established^12^,^13^.
4. Methods for the de novo chemical synthesis of EC metabolites have been developed, allowing for the unambiguous synthesis of authentic standards for analytical method development and validation^14^,^15^,^16^.
5. While widely investigated, the mechanisms of actions of EC are currently unknown, partly because only very few studies have investigated the biological effects of EC metabolites as systemically present in humans^4^.
6. Validated biomarkers for human EC intake are urgently needed, but do not currently exist.
7. Species equivalence of ADME, especially between humans and rodents, is currently assumed, but has not been validated, which represents a serious shortfall, as rodent models are frequently used in investigations aimed at assessing efficacy and safety.

While we and others have made significant advances in understanding the ADME of EC in humans^6^,^17^,^18^,^19^,^20^, the data obtained remain preliminary, exhibiting significant shortfalls and seeming contradictions^6^,^12^,^18^. In the absence of alternative approaches, such as the use of radiolabeled compounds, it is difficult to assess whether or not the reported disparities are merely due to technical limitations and inter-laboratory differences or actually reflect study-specific insights, such as differences in the ADME of EC as a function of the studied population (e.g. sex, age, dietary background, etc.) or the studied EC-containing foods and intake amounts (e.g. food matrix effects, nutrient-nutrient interactions, mastication- and digestibility-related differences in compound liberation). Furthermore, the role of the gut microbiome in the metabolism of EC in humans has only recently been recognized^19^,^21^, increasing the complexity of the EC metabolome.

The current study was based on the oral intake by humans of radiolabeled EC, namely [2-^14^C](−)epicatechin (^14^C-EC; Fig. 1A), allowing for a comprehensive assessment of the ADME of this flavanol. We also focused on gaining novel insights into the role of the human gut microbiome in EC catabolism and, in addition, assessed the important question of species-dependent metabolic equivalence between humans and rodents. The outcomes of the study, which was conducted in the context of a standardized dietary background exposure to flavanols and their polymeric derivatives, the procyanidins, are intended to provide detailed insights into qualitative and quantitative aspects of EC ADME, which will help address current gaps and inconsistencies. Last, but not least, the data generated will be of value in support of efforts aimed at identifying EC-mediated mechanisms of action that underlie its protective effects.

## Results

### Study participants

Eight male volunteers were selected to participate in the ^14^C-EC ADME study. The basic biometric characteristics of the study group were: age, 31 ± 3 y; body weight, 75 ± 11 kg; body-mass index, 24.5 ± 3.3 kg/m^2^; and systolic/diastolic blood pressure of 125 ± 11 over 74 ± 8 mm Hg. No adverse events related to the study treatment were recorded (Supplementary Table S1) and no significant clinical changes were noted from clinical laboratory evaluations, physical examinations and 12-lead electrocardiograms.

### Oral administration of EC

**Figure 1:**
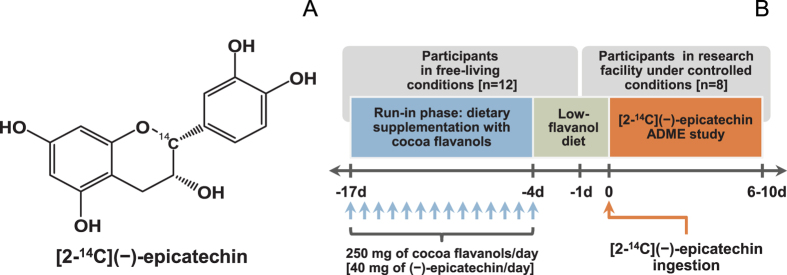
(**A**) Chemical structure of [2-^14^C](−)-epicatechin. (**B**) Schematic representation of study design.

Following the run-in phase (Fig. 1B), participants orally ingested 50 mL of a ^14^C-EC-containing test drink, which delivered 60 mg (207 μmol) of EC, and 300 μCi of radioactivity. Participants were discharged from the research facility 6 to 10 days (mean 8 ± 1 day) after the intake of the ^14^C-EC test drink, when radioactivity excreted in urine and feces over 24 h was <1% of the amount administered.

### Total radioactivity in circulation

**Figure 2:**
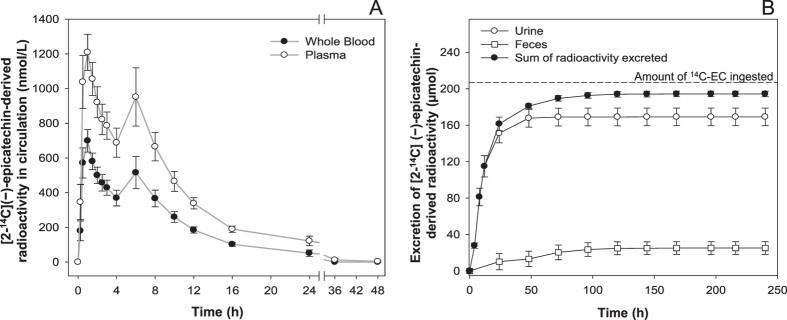
(**A**) Concentration in whole blood and plasma of [2-^14^C](−)-epicatechin -derived radioactivity as a function of time. Data expressed in nM as mean values ± SEM (n = 8). (**B**) Total [2-^14^C](−)-epicatechin -derived radioactivity in urine and feces as a function of time. Data are expressed in μmol as mean values ± SEM (n = 8).

Radioactivity was detected in the circulation 15 min after consumption (Fig. 2A). In whole blood, 2 radioactivity maxima were apparent at 1 h and at 6 h after ^14^C-EC consumption, with peak concentrations (*C*_*max*_) of 699 ± 65 nM and 516 ± 93 nM, respectively. Levels of radioactivity in whole blood fell below the limit of quantitation for all volunteers by 36 h post-consumption. Overall, the total amount of radioactivity in the circulatory system at *C*_*max*_ represented <2% of the amount of ^14^C-EC ingested (Supplementary Fig. S1). Similar to the findings in whole blood, plasma also exhibited 2 radioactivity maxima at 1 h and at 6 h post-intake, with respective *C*_*max*_ values of 1209 ± 104 nM and 952 ± 168 nM. Levels of radioactivity in plasma fell below the limit of quantitation for all volunteers by 72 h post-consumption. The total radioactivity present in whole blood was associated, almost exclusively, with plasma.

### Elimination and mass balance

The elimination of ^14^C-EC-derived radioactivity was investigated in urine and feces (Fig. 2B). Accumulated radioactivity equivalent to 82 ± 5% of the amount ingested was eliminated in urine, with the majority being excreted within the first 24 h after intake. Accumulated radioactivity voided in feces accounted for 12 ± 3% of the ingested ^14^C-EC. Overall, radioactivity in urine and feces accounted for 95 ± 1% of intake, with 5 ± 1% that was not recovered in the collected samples.

### Metabolite profiling

HPLC-MS^2^ coupled with an on-line radioactivity detector, identified and quantified >20 ^14^C-EC-derived metabolites in urine, which were classified in 1) structurally related EC metabolites (SREM), corresponding to *O*-glucuronidated, sulfated and *O*-methylated conjugates bearing an intact flavanol ring and represented mainly by (−)-epicatechin-3′-*O*-β-D-glucuronide (E3′G), (−)-epicatechin-3′-sulfate (E3′S), 3′-*O*-methyl-(−)-epicatechin-5-sulfate (3′ME5S) and 3′-*O*-methyl-(−)-epicatechin-7-sulfate (3′ME7S); 2) 5-carbon side chain ring fission metabolites (5C-RFM), corresponding to *O*-glucuronidated and sulfated derivatives of 5-(3′,4′-dihydroxyphenyl)-γ-valerolactone (γVL), and 5-(3′,4′-dihydroxyphenyl)-γ-hydroxyvaleric acid (γOHVA), represented principally by 5-(4′-hydroxyphenyl)-γ-valerolactone-3′-sulfate (γVL3′S); and 3) 3- and 1-carbon-side chain ring fission metabolites (3/1C-RFM), mainly in the form of 3-(3′-hydroxyphenyl)hydracrylic acid, and the glycinated conjugates of benzoic acids, hippuric acid and 3′-hydroxyhippuric acid (Table 1).

All of these EC-derived compounds were excreted in urine at different times. SREMs were the predominant urinary metabolites collected up to 4 h post-EC intake, whereas 5C-RFMs were the major compounds present from 4 to 12 h after intake. Finally, 3/1-C-RFMs became the main components in urine from 12 h onwards, until radioactivity fell below the limit of detection (Table 1).

**Figure 3:**
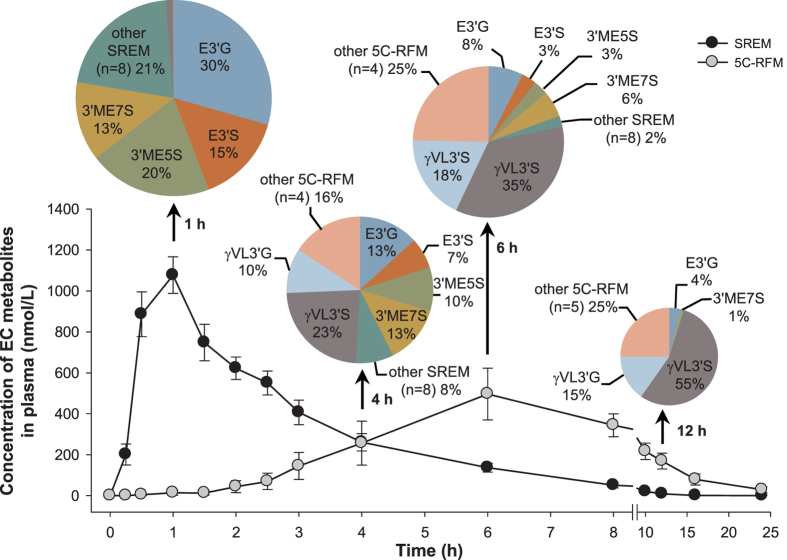
Concentration in plasma of [2-^14^C](−)-epicatechin metabolites as a function of time. SREM: structurally-related (−)-epicatechin metabolites. 5C-RFM: 5-carbon ring fission metabolites. Data are expressed as mean values in nM ± SEM (n = 8). *Insert*: pie charts depict the relative amount (% of total) of individual SREM and 5C-RFM present in plasma at 1 h, 4 h, 6 h and 12 h after ^14^C-EC ingestion (n = 8).

Consistent with the findings from urine, the main SREMs in plasma were identified as E3′G, E3′S, 3′ME5S and 3′ME7S (Table 2, Fig. 3). The SREMs had a combined *C*_*max*_ of 1223 ± 104 nM ~1.0 h after intake (Table 2). Subsequently, they declined rapidly and had all but disappeared from the circulatory system 8 h after ^14^C-EC ingestion (Fig. 3).

5C-RFMs were also detected in plasma but had different pharmacokinetic profiles than SREMs, having a delayed time to reach peak plasma concentration (*T*_*max*_) of ~6 h and longer elimination half-life (*T*_*1/2*_) values that ranged from 3.1–7.6 h (Table 2). The major 5C-RFM detected in plasma was γVL3′S, which was still present at a concentration of ~50 nM, 24 h after ingestion. Due to the low levels of radioactivity, 3/1C-RFMs were not assessed in plasma.

### Species-dependent differences in the circulating profile of SREMs

**Figure 4:**
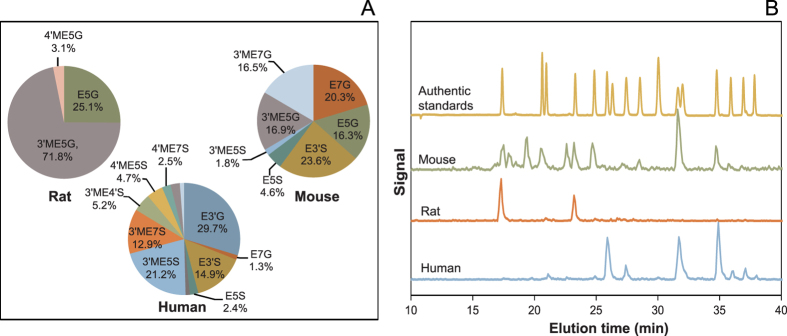
Species-dependent differences in [2-^14^C](−)-epicatechin metabolism. (**A**) Pie charts depict the relative amount (% of total) of individual SREM present plasma at 30 min post ^14^C-EC intake (mouse, rat) and at 1 h post ^14^C-EC intake (human). (**B**) Representative HPLC chromatograms of (yellow) SREM authentic standards (absorbance detection at 280 nm); (green) mouse-specific SREM (on-line radioactivity detection); (red) rat-specific SREM (on-line radioactivity detection); and (blue) human-specific SREM (on-line radioactivity detection).

Thirty min after intragastric administration of ^14^C-EC to Sprague-Dawley rats (2 mg/kg BW; ~10.6 μCi/rat, n = 6), 2 major SREMs appeared in the circulatory system. They were 3′-*O*-methyl(−)-epicatechin-5-*O*-β-D-glucuronide (3′ME5G) and (−)-epicatechin-5-*O*-β-D-glucuronide (E5G) (Fig. 4). No unmetabolized EC and sulfated SREMs were detected.

C57BL/6 mice were also intragastrically administered with ^14^C-EC (2 mg/kg BW; ~6.7 μCi/mouse, n = 6) and, after 30 min, various SREMs, including E3′S, E5G, 3′ME5G, (−)-epicatechin-7-*O*-β-D-glucuronide (E7G) and 3′-*O*-methyl(−)-epicatechin-7-*O*-β-D-glucuronide (3′ME7G), were detected in plasma (Fig. 4). No unmetabolized EC was detected.

## Discussion

**Figure 5:**
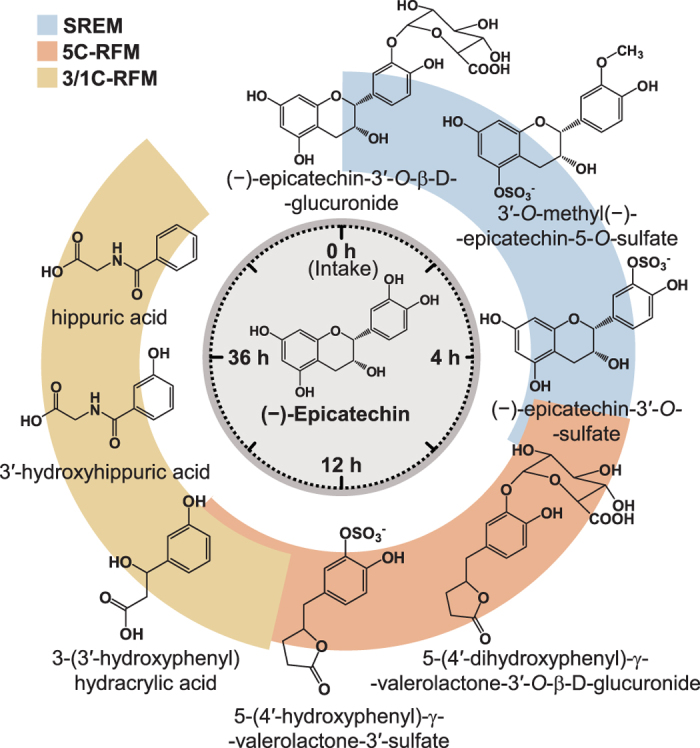
Schematic representation of (−)-epicatechin metabolism in humans as a function of time post-oral intake. SREM: structurally related (−)-epicatechin metabolites. 5C-RFM: 5-carbon ring fission metabolites. 3/1C-RFM: 3- and 1-carbon-side chain ring fission metabolites. The structures of the most abundant (−)-epicatechin metabolites present in the systemic circulation and in urine are depicted.

Based on the use of the radiolabeled ^14^C-EC, we investigated the ADME of this flavanol in humans, under conditions relevant for interpretation of the outcomes in the context of dietary intake and nutrition. In summary:

1. EC is very well absorbed following oral intake in humans. The total 0–48 h recovery of radioactivity in urine accounts for 82 ± 5% of intake, whereas combined recovery in urine and feces represents 95 ± 1% of the ingested ^14^C-EC (Fig. 2B).
2. Extensive biotransformation gives rise to >20 metabolites in plasma and urine (Tables 1 and 2, Fig. 3), with levels of unmetabolized EC below the limits of detection.
3. 20 ± 2% of the radioactivity detected in urine, which must have passed through the systemic circulation, was attributable to SREMs (Table 1).
4. ≈70% of the ingested radioactivity was absorbed in the lower intestine, following catabolism by the gut microbiome that produced a series of RFMs, 42 ± 5% being metabolites of γVL and γOHVA and 28 ± 3% as derivatives of hippuric- and phenolic acids (Table 1).
5. Based on assessments of the SREMs in humans and two rodent species (Fig. 4), we cannot confirm species equivalence for EC metabolism.
6. Excepting effects exerted within the GI tract, the data presented here (Figs 3, 4, 5) make an unequivocal case for the need to test EC metabolites rather than native EC or EC-containing food extracts when aiming to investigate *in vitro* the molecular mechanisms that underlie EC bioactivities observed *in vivo*.

This study was conducted based on a defined exposure to dietary flavanols and procyanidins. The daily intakes by participants of EC, flavanol monomers, and procyanidins with a degree of polymerization (DP) of 2–10 prior to radiotracer administration were 40 mg, 45 mg, and 205 mg, respectively. In comparison, key dietary intervention studies using flavanol-containing foods provided an intake of EC ranging from 90–203 mg/day and procyanidins (DP 2–10) from 390–746 mg/day^7^,^8^,^9^,^10^,^11^. The habitual population-based intake of flavanol monomers (catechin/epicatechin) is reported as averaging 24 or 45 mg per day^22^,^23^, indicating that the EC intake investigated here (60 mg) is relevant and directly applicable in the context of habitual dietary intake and clinical dietary investigations.

The pharmacokinetic data for plasma presented in Table 2 and summarized in Fig. 5 indicate that the major SREMs are E3′G, E3′S, 3′ME5S and 3′ME7S (Figs 3 and 5). These metabolites exhibited *T*_*max*_ values ranging from 0.8 –1.4 h after ingestion of EC, indicative of absorption in the duodenum. SREMs were removed from the circulatory system via urinary excretion, with plasma *T*_*1/2*_ ranging between 1.1 and 2.0 h (Table 2). SREMs were absent from plasma after 6–8 h, but at this point the ingested EC had progressed down the GI tract and reached the colon. There, the microbiota induced opening of the C-ring, resulting in the formation of 5C-RFMs that, after phase II metabolism by enzymes arguably present in the wall of the colon and/or the liver, were represented by a mixture of sulfates and glucuronides of γVLs and γOHVAs that were detected in plasma and urine (Tables 1 and 2;Fig. 3). Most of these colon-derived metabolites remained in the circulatory system for an extended period of time (Table 2, Fig. 3). Overall, the results presented provide an accurate qualitative and quantitative description of the ADME of EC in humans.

Interestingly, analysis of radioactivity in whole blood and plasma (Fig. 2A) demonstrated that the pool of ^14^C-EC metabolites is almost exclusively associated with plasma and not with cellular components of the blood. This finding answers a long-standing question regarding the interpretation of plasma level measurements in the context of assessing the total pool of circulating EC metabolites that may be present in cellular compartments of whole blood. Investigators have described the active transport and accumulation of γVL into erythrocytes and have suggested that GLUT-1 facilitates this transport^24^. However, our data do not confirm the presence of ^14^C-γVL in cellular components of whole blood, but instead identify significant levels of sulfated and glucuronidated γVL metabolites in plasma, indicating that exposure of erythrocytes to native polyphenols or metabolic precursors *ex vitro* may lead to artifacts.

The timing of the appearance of the various radiolabeled compounds in urine over the 48-h period after ingestion of ^14^C-EC was in keeping with the plasma pharmacokinetic profiles that distinguished between components absorbed in the proximal GI tract and the colon. The accumulation of the metabolites in urine also provided quantitative information over and above that gleaned from plasma pharmacokinetic profiles (Tables 1 and 2). The total 0–48 h recovery of the individual radiolabeled metabolites in urine was 185.7 ± 16.3 μmoles of the ingested 207 μmoles of ^14^C-EC (Table 1). This is a recovery of 90 ± 8% compared with an estimate of 82 ± 5% based on liquid scintillation counting of the radioactivity in the unprocessed urine. These two estimates, which are not significantly different considering inter-individual differences, show that we can account for the great majority, if not all, of the EC metabolites that were systemically present in humans. In this context, we did not detect any oxidation products of EC, including ortho-quinones or any quinone-related adducts or derivatives of EC^25^,^26^. This finding represents a compelling argument against the common view that the systemic biological effects mediated by EC are predominantly based on direct antioxidant mechanisms involving hydrogen-/single electron transfer.

The urinary data summarized in Table 1 reveal that EC metabolites absorbed in the small intestine correspond to 20 ± 2% of intake, a figure that is in agreement with previous, albeit less precise, estimates of urinary excretion following ingestion of supplements containing a mixture of flavanols^6^. Most EC absorption occurred in the colon, with γVL and γOHVA metabolites being excreted in amounts corresponding to 42 ± 5% of intake. In addition, there was 28 ± 3% excretion of phenolic acids and hippuric acid metabolites (Table 1). Thus, in total, 70% of the ingested ^14^C-EC was absorbed into the circulatory system via the colon compared to 20% being absorbed from the small intestine. This finding highlights the importance of the colonic microbiome as a key factor for the absorption and metabolic fate of EC in the human body.

Our radiotracer-based data validate and significantly extend recent data on the identification of human EC metabolites obtained using standard ^12^C-EC-based approaches^18^. Also, recent findings by Actis-Goretta *et al*., which were not obtained using isotopically labeled compounds, can largely be confirmed in this context^20^. However, Actis-Goretta *et al*., in a study of 5 individuals, reported the presence of (−)-epicatechin-4′-β-D-glucuronide and (−)-epicatechin-4′-sulfate that could not be identified by us, even though the use of a radiolabeled tracer should have enabled the detection of these compounds. While this discrepancy may be explained by inter-individual differences in EC metabolism, we have not observed such variations in EC metabolism in the present study, and it seems more likely that the compounds reported as EC metabolites by Actis-Goretta *et al*.^20^ are in fact metabolites of the EC stereoisomer, (−)-catechin.

Variations in the published *T*_*max*_ values for EC metabolites range from 0.8 h to 3.8 h^6^,^18^,^20^ and are most likely a consequence of differences in food matrix-specific effects, as the nature of the food matrix impacts mastication, gastric emptying, digestibility, etc., and therefore, the liberation of EC, and any other food constituent for that matter, from the ingested food. To exclude these potential confounders from this investigation, we chose to use a simple water matrix as the vehicle for EC ingestion. Nevertheless, questions related to the role of the food matrix in the ADME and *T*_*max*_ of EC metabolites under real-life conditions remain pertinent from a nutritional perspective and should also be considered in the context of designing clinical dietary intervention studies.

While γVLs generally have been identified previously as gut microbiome-derived flavanol metabolites in humans^27^,^28^, the data presented here provide a deeper understanding with regard to the identity as well as the pharmacokinetics of specific EC-derived γVL metabolites present in humans. Considering factors such as 1) concentrations in urine and plasma; 2) *T*_*1/2*_; 3) specificity; 4) chemical stability; 5) temporality; and 6) consistency of observed occurrence^29^, γVL metabolites represent far better candidates than SREMs for the development and validation of objectives biomarkers for the dietary intake of EC and potentially, also procyanidins and other flavanol monomers^19^. Future research may also be able to utilize stereochemistry-specific γVL analyses to further increase the specificity of flavanol-/procyanidin-intake assessments.

The data depicted in Fig. 4 represent initial insights into the question of whether or not EC metabolism in humans is equivalent to that occurring in other mammals, in this case, rodents. While this question has relevance from a purely scientific perspective, as rodent models are frequently used to study the pharmacodynamic effects of drugs and nutrients *in vivo*, it also has regulatory relevance, especially with regard to the assessment of safety risks and potential toxicological effects in the context of drug and food ingredient safety. Although limited by the fairly small numbers of individuals investigated, and by assessing only the SREM-group of EC-derived compounds, our data demonstrate considerable species-dependent differences in EC metabolism. Despite exhibiting similar metabolic themes, i.e., glucuronidation, sulfation, and methylation across species, the metabolism of EC, as judged by the profile of SREMs, is very different between humans and rats and, to a somewhat lesser degree, between humans and mice (Fig. 4). Of the 4 major SREMs present in humans, accounting for ≈80% of all SREM detected in humans, none was detected in rats, and only one of the SREMs detected in rats, namely 3′ME5G, was present in humans, albeit in low abundance (<3% of total SREM) (Fig. 4.). While more complex due to the higher number of SREMs detected, the situation in mice is similar, although perhaps less pronounced; of the 4 major SREMs present in humans, only two, E3′S and 3′ME5S, were detected in mice, representing ≈25% of all mouse SREMs identified (Fig. 4). Common principles of medicinal chemistry may predict that individual SREMs, while similar in chemical structure, would nevertheless have significantly different effects when interacting with any given target protein. However, in the absence of a confirmed mechanism of action or target protein, it is at present not possible to test this hypothesis. The data obtained here are preliminary to the extent that it is not possible to assess to what degree the ADME of EC varies as a function of the specific strain of rat or mouse and the intake level tested. Nevertheless, while further research is required, this finding should be carefully considered when evaluating the limitations, and thus the relevance, of rodent-based testing of the safety and efficacy of flavanols and indeed of other polyphenolic compounds for that matter. This is of particular relevance when considering recent reports regarding the safety of phytochemical-containing dietary supplements intake^30^.

Taken together, the data presented here provide detailed radiotracer-based insights into the ADME of EC in humans, which highlight the wider-ranging consequences of the interpretation of past and present data on the bioactivity of dietary flavanols in the context of epidemiological and clinical dietary intervention studies, as well as *in vitro* investigations based on cell culture systems. In addition, this investigation provides insights relevant for assessing the safety of EC and diets rich in flavanols, such as information for considering potential nutrient-drug interactions, as well as insights into potential limitations of rodent-based safety assessments. We hope that the outcomes of this investigation will also be of utility in guiding the design of future dietary intervention studies. It is also apparent that research using *in vitro* cell culture test systems should refrain from using flavanol-containing food extracts and unmetabolized EC and exercise caution when considering direct antioxidant mechanisms in the context of understanding the mode of action of flavanols *in vivo*. Considering this investigation from a broader perspective, it is evident that basic insights into the ADME of bioactives are of fundamental importance, and a greater appreciation and recognition of the practical relevance of ADME research in general would be of considerable benefit to many aspects of nutritional and biomedical research.

## Methods

### Chemicals

Authentic, de novo chemically synthesized EC metabolite standards (SI Materials and Methods), were synthesized and characterized as described elsewhere^14^,^15^,^16^ and supplied by the Institute of Pharmaceutical Discovery, LLC (Branford, CT). EC was provided by Mars Inc. (Hackettstown, NJ, USA). ^14^C-EC (Fig. 1A) was de novo synthesized by Quotient Bioresearch Ltd. (Cardiff, UK) according to Sharma *et al*.^31^ and tested and cleared for human use in this study. Other reagents were purchased from Fisher (Pittsburgh, PA).

### Participants

We screened healthy, male volunteers between 18 and 50 years of age, with a body weight between 60 and 100 kg, and a body mass index between 19 and 30 kg/m^2^, as detailed in Supplementary Methods.

### Human study design

The study was a single-center, open-label, and non-randomized study with two distinct elements: a run-in phase to establish a more defined dietary background of dietary flavanols and procyanidins and the subsequent ^14^C-EC ADME study (Fig. 1B). Participants (n = 12) were enrolled in the run-in phase (day –17 to day –4) and asked to supplement their daily diet for 14 days with a commercially available flavanol-containing cocoa-based drink (250 mg cocoa flavanols; 40 mg of EC). This was followed by a 4-day period (day –4 to 0) of a low-flavanol diet. One day (study day -1) prior to the initialization of the ^14^C-EC ADME study, all eligible participants were accommodated under controlled conditions at a metabolic research facility (Covance Clinical Pharmacology Inc., Wisconsin) until completion of the study. Based on the compliance of the first part of the study, 8 participants were selected to participate in the ^14^C-EC ADME study. During the ^14^C-EC ADME study (Fig. 1B.), volunteers received a low-flavanol, standardized high-fiber diet, and were encouraged to maintain an adequate level of hydration (water *ad libitum*). Volunteers were asked to fast for 8 h before the initiation of the study and for at least 4 h after the intake of the ^14^C-EC test drink (water *ad libitum*). Venous blood samples were taken using Vacutainer collection tubes, containing potassium EDTA at 0 h (just prior to ^14^C-EC ingestion), and at 0.25, 0.5, 1, 1.5, 2, 2.5, 3, 4, 6, 8, 10, 12, 16, 24, 36, and 48 h after test drink consumption. Plasma was obtained by whole blood centrifugation at 2000 × *g* for 15 min at 5 °C, separated into aliquots and stored at −80 °C until analysis. All urine and feces from the time just prior to the consumption of the test drink were collected at different time intervals for radioactivity and metabolite profiling. Clinical parameters and laboratory evaluations of participants were assessed at screening, on study day –1, and after completing the study, as detailed in Supplementary Methods.

### Research ethics

The study protocol was approved by the Independent Institutional Review Board (Sunrise, Florida) in accordance with the United States Code of Federal Regulations governing Protection of Human Subjects (21 CFR 50), Financial Disclosure by Clinical Investigators (21 CFR 54), and IRB (21 CFR 56). In addition, this study was conducted under a Radioactive Drug Research Committee-approved protocol, in accordance with 21 CFR 361.1. All subjects gave their written informed consent to participate. This study was registered at clinicaltrials.gov (NCT01969994) in October 2013.

### Run-in phase flavanol-containing drink

The drink consisted of a commercially available flavanol- and procyandin-containing, cocoa-based drink mix powder (CocoaVia, Mars Inc.) that was mixed with 240 mL of either water or milk (1% fat) and consumed once daily. Per serving, the drink provided 250 mg of cocoa flavanols, including 40 mg of EC and 205 mg of procyanidins (DP 2–10).

### ^14^C-EC test drink

The 50-mL drink consisted of an aqueous solution of ^14^C-EC (specific activity 14.5 μCi μmol^−1^) and delivered 60 mg (207 μmol) of EC and 300 μCi (660 × 10^6 ^dpm) of radioactivity. The drink was prepared one day before consumption to verify correctness of composition and stability. Volunteers received the ^14^C-EC test drink (50 mL), followed by 3 × 50 mL of water that was used to rinse the test drink container, and 40 mL of water. The radioactivity remaining in the test drink containers was measured, and the residual radioactivity recovered from each container was subtracted from the amount administered to the corresponding volunteer.

### Measurement of total radioactivity

Radioactivity in aliquots of plasma and urine was measured by liquid scintillation counting. Blood and feces were dried and oxidized by combustion using a 307 Sample Oxidizer (Packard Instrument Company, Meriden, NH) before being analyzed by liquid scintillation counting.

### Metabolite profiling and quantification

Samples were processed as described previously^18^ and analyzed by HPLC-MS^2^ in conjunction with an on-line HPLC-radioactivity monitor as described in SI Materials and Methods. Identification of metabolites involved comparing the retention time with authentic standards and MS^2^ fragmentation patterns (Supplementary Methods). Quantification of metabolites in urine was based on HPLC radioactivity peak area (limit of quantification: 10^3^ dpm). Due to the low levels of radioactivity, quantification in plasma was based on targeted HPLC-MS^2^ using standard curves obtained with authentic standards (Supplementary Methods).

### Rodent study

All procedures involving rats and mice were approved by the Institutional Animal Care and Use Committees at Covance Laboratories (Madison, WI, USA) and the University of California Davis. Both institutions are accredited by AAALAC International (Association for Assessment and Accreditation of Laboratory Animal Care International). All procedures involving rats and mice were conducted in accordance with guidelines from the Animal Care and Use Committees at Covance Laboratories (Madison, WI, USA) and the University of California Davis conformed with the US Government Animal Welfare Act (7 U.S.C. § 2131) and the Health Research Extension Act (Public Law 99–158).

To assess the profile of SREMs in rodents, Sprague-Dawley rats (male, 9–10 week old, 270 ± 16 g of BW) and C57BL/6 mice (20 ± 1 g) were administered by gavage 2 mg/kg BW of ^14^C-EC (corresponding to ≈10.6 μCi/rat and 6.7 μCi/mouse). Blood samples were collected 30 min after administration. Plasma was obtained by centrifugation at 4 °C for 15 min at 1800x*g*, separated in aliquots and analyzed by HPLC as described previously^18^ with an on-line radioactivity detector (Radiomatic 610TR Flow System Analyzer, PerkinElmer, Inc., Waltham, Mass).

### Pharmacokinetic analysis

Data are expressed as mean values ± standard error, or as otherwise specified. Pharmacokinetic parameters were calculated using non-compartmental analysis with WinNonlin (Pharsight Corporation, Version 5.2).

## Additional Information

**How to cite this article**: Ottaviani, J. I. *et al*. The metabolome of [2-^14^C](−)-epicatechin in humans: implications for the assessment of efficacy, safety, and mechanisms of action of polyphenolic bioactives. *Sci. Rep.* **6**, 29034; doi: 10.1038/srep29034 (2016).

## Supplementary Material

Supplementary Information

## Figures and Tables

**Table 1:** Human [2-^14^C](−)-epicatechin metabolites present in urine.

[2-^14^C](−)-Epicatechin metabolites	Urine collection periods					
	0–4 h	4–8 h	8–12 h	12–24 h	24–48 h	0–48 h
(−)-Epicatechin-3′-*O*-glucuronide	8.0 ± 1.4	5.7 ± 1.3	2.9 ± 0.8	2.0 ± 0.8	–	18.6 ± 2.5
(−)-Epicatechin-3′-sulfate	6.0 ± 0.7	1.2 ± 0.4	0.3 ± 0.1	0.2 ± 0.2	–	7.7 ± 0.9
(−)-Epicatechin-5-sulfate	1.3 ± 0.2	–	–	–	–	1.3 ± 0.2
3′-*O*-Methyl(−)-epicatechin-4′-sulfate	0.7 ± 0.2	–	–	–	–	0.7 ± 0.2
3′-*O*-Methyl(−)-epicatechin-5-sulfate	7.1 ± 0.9	1.8 ± 0.3	0.4 ± 0.1	0.3 ± 0.2	–	9.6 ± 1.2
3′-*O*-Methyl(−)-epicatechin-7-sulfate	0.9 ± 0.2	0.3 ± 0.1	–	–	–	1.2 ± 0.3
4′-*O*-Methyl(−)-epicatechin-5-sulfate	1.0 ± 0.2	0.1 ± 0.1	–	–	–	1.1 ± 0.3
4′*-O*-Methyl(−)-epicatechin-7-sulfate	0.3 ± 0.1	0.1 ± 0.1	–	–	–	0.4 ± 0.2
**Sum of SREMs**	**25.3 ± 3.1**	**9.2 ± 0.9**	**3.6 ± 0.8**	**2.5 ± 0.8**	**–**	**40.6 ± 4.5** ***(20 ± 2%)***
5-(Phenyl)-γ-valerolactone-sulfate-*O*-glucuronide-I	0.1 ± 0.1	0.9 ± 0.8	0.2 ± 0.2	0.4 ± 0.3	–	1.6 ± 0.7
5-(4′-Hydroxyphenyl)-γ-valerolactone-3′-sulfate	3.6 ± 1.1	16.0 ± 3.0	9.8 ± 2.0	8.1 ± 2.9	0.5 ± 0.5	38.0 ± 4.7
5-(Phenyl)-γ-valerolactone-3′-sulfate	0.1 ± 0.1	2.4 ± 1.1	1.2 ± 0.9	1.0 ± 0.8	–	4.7 ± 2.7
5-(3′-Hydroxyphenyl)-γ-hydroxyvaleric acid-4′-sulfate; 5-(3′-Hydroxyphenyl)-γ-valerolactone-4′-*O*-glucuronide	0.2 ± 0.2	7.7 ± 1.5	3.7 ± 1.2	2.3 ± 1.2	–	13.9 ± 2.2
5-(4′-Hydroxyphenyl)-γ-hydroxyvaleric acid-3′-sulfate; 5-(4′-Hydroxyphenyl)-γ-valerolactone-3′-*O-*glucuronide; 5-(Phenyl)-γ-valerolactone-sulfate-*O*-glucuronide-II	0.9 ± 0.6	13.1 ± 4.5	6.6 ± 1.3	3.4 ± 1.3	–	24.0 ± 5.2
5-(3′-Hydroxyphenyl)-γ-hydroxyvaleric acid-4′-*O*-glucuronide	–	1.3 ± 0.5	0.5 ± 0.3	0.5 ± 0.1	–	2.3 ± 0.7
5-(Phenyl)-4-hydroxyvaleric acid-3′-sulfate	–	1.9 ± 1.0	0.3 ± 0.2	0.7 ± 0.7	–	2.9 ± 1.6
**Sum of 5C-RFMs**	**4.9 ± 1.6**	**43.3 ± 8.3**	**22.3 ± 4.1**	**16.4 ± 5.4**	**0.5 ± 0.5**	**87.4 ± 9.6** ***(42***** ± *****5%)***
Hydroxyphenylacetic acid-sulfate	–	0.9 ± 0.5	0.8 ± 0.6	1.2 ± 0.4	–	2.9 ± 1.1
3-(3′-Hydroxyphenyl)hydracrylic acid	–	0.7 ± 0.4	2.2 ± 0.5	5.3 ± 2.3	3.3 ± 1.7	11.5 ± 4.4
Hippuric acid	0.6 ± 0.4	7.2 ± 3.9	5.6 ± 2.6	7.1 ± 2.1	5.9 ± 4.1	26.4 ± 7.1
3′-Hydroxyhippuric acid	–	1.6 ± 0.5	2.8 ± 0.7	7.0 ± 2.9	5.5 ± 2.3	16.9 ± 5.8
**Sum of 3/1C-RFMs**	**0.6 ± 0.4**	**10.4 ± 4.1**	**11.4 ± 2.5**	**20.6 ± 4.1**	**14.7 ± 5.5**	**57.7 ± 6.9** ***(28***** ± *****3%)***

Data are expressed as mean ± SEM in μ mol (n = 8); bold, italicized figures in parentheses represent urinary recoveries of metabolites as a percentage of (—)-epicatechin intake; SREMs-structurally related (—)-epicatechin metabolites; 5C-RFMs-5 carbon side chain ring fission metabolites; 3/1C-RFMs-3 to 1 carbon side chain ring fission metabolites;-not detected.

**Table 2:** Pharmacokinetic parameters of human [2-^14^C](−)-epicatechin metabolites present in plasma.

[2-^14^C](−)-Epicatechin metabolites *(number of isomers)*	*C*_*max*_ (nM)	*T*_*max*_ (h)	*AUC*_*0-24h*_ (nM/h)	*T*_*1/2*_ (h)
(−)-Epicatechin-3′-*O*-glucuronide	359 ± 23	0.8 ± 0.1	1624 ± 332	2.0 ± 1.1
(−)-Epicatechin-7-*O*-glucuronide†	22 ± 7	0.8 ± 0.1	45 ± 14	1.7 ± 0.3
(−)-Epicatechin-3′-sulfate	191 ± 17	1.1 ± 0.1	633 ± 135	2.0 ± 0.3
(−)-Epicatechin-5-sulfate	32 ± 4	0.8 ± 0.1	92 ± 25	1.9 ± 0.3
(−)-Epicatechin-7-sulfate†	19 ± 3	1.1 ± 0.2	43 ± 16	2.2 ± 0.4
3′-*O*-Methyl-(−)-epicatechin-4′-sulfate	53 ± 13	0.9 ± 0.1	203 ± 69	2.3 ± 0.6
3′-*O*-Methyl-(−)-epicatechin-5-sulfate	240 ± 24	0.9 ± 0.1	915 ± 178	1.6 ± 0.1
3′-*O*-Methyl-(−)-epicatechin-7-sulfate	159 ± 20	1.1 ± 0.1	1026 ± 260	2.4 ± 0.2
4′-*O*-Methyl-(−)-epicatechin-5-sulfate	53 ± 14	0.9 ± 0.1	128 ± 45	1.4 ± 0.3
4′-*O*-Methyl-(−)-epicatechin-7-sulfate	33 ± 5	1.4 ± 0.2	166 ± 54	2.6 ± 0.5
3′-*O*-Methyl-(−)-epicatechin-5-*O*-glucuronide^†^	23 ± 5	0.8 ± 0.2	25 ± 10	1.3 ± 0.2
3′-*O*-Methyl-(−)-epicatechin-7-*O*-glucuronide†	39 ± 5	0.9 ± 0.1	77 ± 31	1.3 ± 0.3
**Sum of SREMs**	**1223 ± 104**	**1.0 ± 0.1**	**4943 ± 471**	**1.9 ± 0.1**
5-(4′-Hydroxyphenyl)-γ-valerolactone-3′-sulfate	272 ± 56	6.4 ± 1.0	7595 ± 2684	6.3 ± 1.7
5-(3′-Hydroxyphenyl)-γ-valerolactone-4′-*O*-glucuronide	52 ± 9	6.1 ± 0.8	1329 ± 396	4.4 ± 1.3
5-(4′-Hydroxyphenyl)-γ-valerolactone-3′-*O*-glucuronide	125 ± 30	6.8 ± 0.8	1908 ± 787	3.1 ± 0.6
5-(Phenyl)-γ-valerolactone-*O*-sulfate-*O*-glucuronide *(2)*	39 ± 9	5.5 ± 1.1	1017 ± 481	6.4 ± 2.2
5-(Hydroxyphenyl)-γ-hydroxyvaleric acid-sulfate (2)	56 ± 9	5.9 ± 0.6	1492 ± 475	7.6 ± 3.5
5-(3′-Hydroxyphenyl)-γ-hydroxyvaleric acid-4′-*O*-glucuronide	54 ± 14	4.9 ± 1.3	835 ± 355	6.5 ± 0.8
**Sum of 5C-RFMs**	**588 ± 102**	**5.8 ± 0.4**	**14352 ± 2264**	**5.7 ± 0.7**

Data expressed as mean values ± SEM (n = 8).

^†^metabolite detected in plasma but not in urine; *C*_*max*_–peak plasma concentration; *T*_*max*_–time to reach peak plasma concentration; *AUC*_*0–24h*_–area under the curve; *T*_*1/2*_–elimination half-life; SREMs–structurally related (−)-epicatechin metabolites; 5C-RFMs–5 carbon side chain ring fission metabolites.
