# Supplementary material for: The metabolome of [2-14C](−)-epicatechin in humans: implications for the assessment of efficacy, safety, and mechanisms of action of polyphenolic bioactives
Source: Sci Rep. 2016 Jul 1;6:29034. doi: 10.1038/srep29034 (PMC4929566; doi:10.1038/srep29034)
Supplement: Supplementary Information [file srep29034-s1.pdf]

Supplementary Information

The metabolome of [2-14C](−)-epicatechin in humans: implications for the assessment of efficacy, safety, and mechanisms of action of polyphenolic bioactives

Javier I. Ottaviani[1]*, Gina Borges[2], Tony Y. Momma[2], Jeremy P.E. Spencer[3], Carl L. Keen[2], Alan Crozier[2] and Hagen Schroeter[1]

[1]Mars, Inc., McLean, VA 22101, USA

[2]Department of Nutrition, University of California Davis, Davis, CA 95616 USA

[3]Department of Food and Nutritional Sciences, University of Reading, Whiteknights, Reading RG6 6AP,UK.

*To whom correspondence should be addressed. Email: Javier.Ottaviani@mss.effem.com

Supplementary Tables

Supplementary Table S1. Summary of adverse events (AE). There were 6 adverse events reported by 3 different participants. The physician responsible for medical aspects of the study determined that all reported adverse events were mild in severity, and none was deemed related to study treatment.

Type of AE reported	Number of AE reported	Severity	Relationship with the study treatment
Chapped lips	1	mild	Not related
Diarrhea	1	mild	Not related
Venipuncture site Swelling	1	mild	Not related
Vessel puncture site Hematoma	1	mild	Not related
Muscle spasms	1	mild	Not related
Dizziness	1	mild	Not related

Supplementary Figures

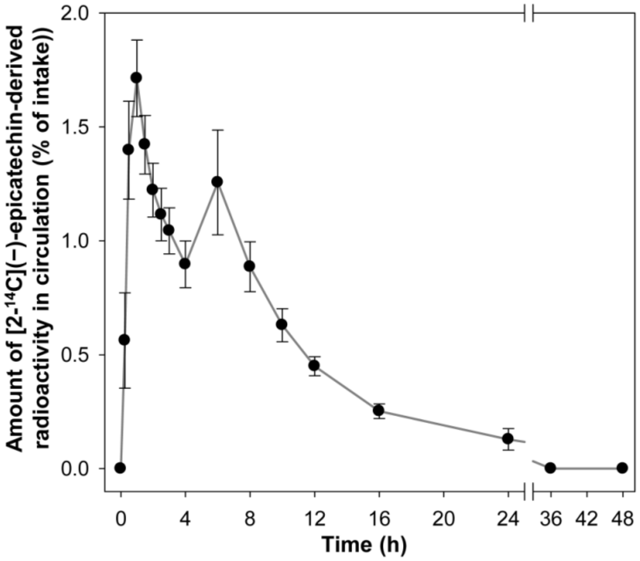

Supplementary Figure S1. Total [2-^{14}C](−)-epicatechin-derived radioactivity in circulation. Amount of [2-14C](−)-epicatechin (^{14}C-EC)-derived radioactivity in circulation as a function of time. Data are expressed as mean values ± SEM [n=8] of the amount of ^{14}C-EC-derived radioactivity present in circulation relative to the amount of ^{14}C-EC consumed, in percentage (%). The amount of ^{14}C-EC-derived radioactivity in circulation was calculated from the concentration of ^{14}C-EC-derived radioactivity in whole blood and the volume of blood for each participant. Volume of blood was calculated using Nadler's equation [1].

Supplementary Methods

Chemicals

Authentic, chemically de novo synthesized (−)-epicatechin metabolite standards, including 5-(3′,4′-dihydroxyphenyl)-γ-valerolactone and the ammonium salts of 5-(3′,4′-dihydroxyphenyl)-γ-valerolactone-3′-sulfate, 5-(3′,4′-dihydroxyphenyl)-γ-valerolactone-4′-sulfate, (−)-epicatechin-4′-sulfate, (−)-epicatechin-3′-sulfate, (−)-epicatechin-5-sulfate, (−)-epicatechin-7-sulfate, (−)-epicatechin-4′-*O*-β-D-glucuronide, (−)-epicatechin-3′-*O*-β-D-glucuronide, (−)-epicatechin-5-*O*-β-D-glucuronide, (−)-epicatechin-7-*O*-β-D-glucuronide, 3′-*O*-methyl-(−)-epicatechin-4′-sulfate, 3′-*O*-methyl-(−)-epicatechin-5-sulfate, 3′-*O*-methyl-(−)-epicatechin-7-sulfate, 3′-*O*-methyl-(−)-epicatechin-5-*O*-β-D-glucuronide, 3′-*O*-methyl-(−)-epicatechin-7-*O*-β-D-glucuronide, 4′-*O*-methyl-(−)-epicatechin-5-sulfate, 4′-*O*-methyl-(−)-epicatechin-7-sulfate, 4′-*O*-methyl(−)-epicatechin-3′-*O*-β-D-glucuronide, 4′-*O*-methyl-(−)-epicatechin-5-*O*-β-D-glucuronide and 4′-*O*-methyl-(−)-epicatechin-7-*O*-β-D-glucuronide were provided by the Institute of Pharmaceutical Discovery, LLC (Branford, CT). 3-(3′-Hydroxyphenyl)hydracrylic acid was purchased from Medical Isotopes, (Pelham NH). Toronto Research Chemicals (North York, ON) supplied 5-(3′,4′-dihydroxypheny)-γ-hydroxyvaleric acid

Participants

We screened healthy, male volunteers between 18 and 50 years of age, with a body weight between 60 and 100 kg, and a body mass index between 19 and 30 kg/m^2. Exclusion criteria were a history or clinical symptoms of significant metabolic, hematological, pulmonary, cardiovascular, gastrointestinal, neurologic, hepatic, renal, urological, and psychiatric disorders. Based on a dietary/life-style questionnaire, we also excluded volunteers with food allergies, lactose intolerance, a history of stomach or intestinal surgery; users of tobacco products within 6 months prior to study entry; and vegans, vegetarians, and anyone who consumed less than 1 to 2 servings of fruits/vegetables per day. During the study, volunteers received a standardized high-fiber diet at scheduled times that did not conflict with other study-related activities and were encouraged to maintain an adequate level of hydration (water ad libitum). Volunteers were asked to fast for 8 h before the initiation of the study and for at least 4 h after the intake of the [2-^{14}C](−)-epicatechin test drink (water ad libitum).

Clinical Parameters and Laboratory Evaluation

Clinical parameters determined included oral temperature, respiratory rate, automated seated blood pressure, pulse, and 12-lead electrocardiogram. Laboratory evaluation included the assessment of cell blood count and metabolic, kidney and liver panel in blood and plasma, respectively. Clinical parameters and laboratory evaluations were assessed at screening, on study day –1, and after completing the study. All samples for clinical laboratory evaluations were analyzed by Meriter Laboratories (Madison, Wisconsin).

Metabolite Profiling and Quantification

Samples were analyzed on a Surveyor HPLC system comprising an autosampler cooled to 4 °C, an HPLC pump and a column oven, maintained at 40 °C, a photodiode array (PDA) detector, scanning from 250 nm to 600 nm (Thermo Finnigan, CA, USA). After

passing through the HPLC column and the flow cell of the PDA detector the column eluate was split and 0.2 mL directed to an LCQ Duo tandem mass spectrometer (Thermo-Finnigan, UK), with an electrospray interface in negative ionization mode. The remaining 0.8 mL of eluate was mixed at a “T” with scintillation cocktail (Optiflow Safe One, Fisons, Loughborough, UK,) pumped at 2.5 mL/min and directed to a radioactivity monitor (Reeve Analytical Model 9701, LabLogic, Sheffield, UK) fitted with a 1.0 mL homogeneous flow cell.

Tuning of the mass spectrometer was optimized by infusing a standard of EC dissolved in the initial HPLC mobile phase into the source at a flow rate of 0.2 mL/min. Capillary temperature was 290°C; sheath and auxiliary gases were 30 and 60 units/min, respectively; source voltage was 4 kV and collision energy was set at 35%. The mass spectrometer was operated either in full scan mode from *m/z* 100 to 600 or selective reaction monitoring (SRM) mode for a number of SREMs and 5C-RFM derivatives (Supplementary Table S2).

Data were processed by Xcalibur software program version 2.1. The quantification of the EC metabolites in plasma was based on calibration curves with de novo chemically synthesized authentic reference standards, and total recoveries were based on the levels of radioactivity measured by scintillation counts present in aliquots of samples prior to HPLC analysis. Due to the absence of standards, the quantification of some 5C-RFMs was based on dilutions of a urine sample that contained ^{14}C-peaks partially identified as a 5-(phenyl)-γ-valerolactone-sulfate-*O*-glucuronide (*m/z* 463), and a 5-(hydroxyphenyl)-γ-hydroxyvaleric acid-sulfate (*m/z* 305) and the corresponding glucuronide (*m/z* 401) metabolite. In this way, calibrations curves were constructed (r=0.9983 – 0.9631), in which the response of the mass spectrometer was related to radioactivity, which in turn equated with nmoles of the metabolites derived exclusively from ^{14}C-EC

Supplementary Table S2. Summary of HPLC-MS analysis of [2-^{14}C](−)-epicatechin metabolites in urine. MSI MI (Metabolite Standards Initiative – Metabolites Identification) level which is a confidence level of the identification where level 1 stands for fully elucidated (i.e. by in-house comparison with a standard or NMR) and level 2 for a well characterized, but not yet fully structurally elucidated metabolite. Since all radiolabeled ring fission products, such as valerolactones, valeric acids and phenolic acids are derived from [2-^{14}C](−)-epicatechin, the 3′- and/or 4′ carbons are the probable positions of hydroxyl groups as well as *O*-methylation, sulfation and glucuronidation. 5-(4′-Hydroxyphenyl)-γ-valerolactone-3′-*O*-glucuronide, 5-(3′-hydroxyphenyl)-γ-valerolactone-4′-*O*-glucuronide, 5-(phenyl)-γ-valerolactone-3′-sulfate and 5-(phenyl)-γ-valerolactone-3′-*O*-β-D-glucuronide, identified by NMR, were isolated from urine collected in a separate study

**Peak No.**	**Rt (min)**	**[M–H]$^-$ (*m/z*)**	**MS^2 ions (*m/z*)**	**Identification/tentative identifications**	**MSI MI level***
**1**	18.4	194	150, 93	3′-Hydroxyhippuric acid	1
**2**	19.5	181	137, 121	3-(3′-Hydroxyphenyl)hydracrylic acid	1
**3**	27.2	401	225	5-(3′-Hydroxyphenyl)- γ-hydroxyvaleric acid-4′-*O*-β-D-glucuronide	2
**4**	29.5	178	135	Hippuric acid	1
**5**	37.6	247	167, 123	Hydroxyphenylacetic acid-sulfate	2
**6**	46.4	463	383, 287	5-(Phenyl)- γ-valerolactone-sulfate-*O*- β-D-glucuronide I	2
**7**	49.0	305<br>383	225, 207<br>207, 163	5-(3′-Hydroxyphenyl)- γ-hydroxyvaleric acid-4′-sulfate<br>5-(3′-Hydroxyphenyl)- γ-valerolactone-4′-*O*-glucuronide	2<br>1
**8**	52.1	305<br>383<br>463	225, 207<br>207, 163<br>383, 287	5-(4′-Hydroxyphenyl)- γ-hydroxyvaleric acid-3′-sulfate<br>5-(4′-Hydroxyphenyl)- γ-valerolactone-3′-*O*- β-D-glucuronide<br>5-(Phenyl)- γ-valerolactone-sulfate-*O*-glucuronide II	2<br>1<br>2
**9**	57.7<br>58.3<br>58.3	479<br>465<br>367	303<br>289<br>191, 175	3′-*O*-Methyl-(−)-epicatechin-7-*O*- β-D-glucuronide<br>(−)-Epicatechin-3′-*O*- β-D-glucuronide<br>5-(Phenyl)- γ-valerolactone-3′-*O*- β-D-glucuronide	1<br>1
**10**	59.1	289	209	5-(Phenyl)- γ-hydroxyvaleric acid-3′-sulfate	2
**11**	67.2	369	289, 245	(−)-Epicatechin-5-sulfate	1
**12**	75.6	287	207, 163	5-(4′-Hydroxyphenyl)- γ-valerolactone-3′-sulfate	1
**13**	81.8	369	289, 245	(−)-Epicatechin-3′-sulfate	1
**14**	86.9	383	303	3′-*O*-Methyl-(−)-epicatechin-4′-sulfate	1
**15**	92.8	271	191, 147	5-(Phenyl)- γ-valerolactone-3′-sulfate	1
**16**	96.4	383	303	3′-*O*-Methyl-(−)-epicatechin-5-sulfate	1
**17**	103.3	383	303	3′-*O*-Methyl-(−)-epicatechin-7-sulfate	1
**18**	108.3	383	303	4′-*O*-Methyl-(−)-epicatechin-5-sulfate	1
**19**	112.7	383	303	4′-*O*-Methyl-(−)-epicatechin-7-sulfate	1

HPLC-MS identifications of radiolabeled [2-^{14}C](−)-epicatechin metabolites in urine

Urine was analyzed with radioactivity and mass spectrometry (MS) detection using a 250 mm x 4.6 mm i.d. 5 µm Luna Phenyl-Hexyl column (Phenomenex, Macclesfield, UK) eluted at a flow rate of 1 mL/min with a 120 min gradient of 5-35% methanol in 0.1 % formic acid. Metabolite identification was performed by 1) co-chromatography of metabolites with reference compounds and a comparison of their MS fragmentation patterns [2], 2) by comparing *m/z* fragmentation patterns with earlier in-house observed metabolites in urine after polyphenol intake [3,4] and 3) by comparing *m/z* fragmentation patterns to databases of metabolites reported in earlier polyphenol feeding studies[5-7].

The basis of the identifications of 28 urinary metabolites is summarized in Supplementary Table S2, which also provides information on the MSI MI (Metabolite Standards Initiative – Metabolites Identification) level, a confidence level for identification where level 1 stands for fully elucidated (i.e., by comparison with a standard) and level 2 for a well characterized, but not yet fully structurally elucidated metabolite [8]. Since all radiolabeled ring fission products such as valerolactones, valeric acids and phenolic acids are derived from [2-^{14}C](−)-epicatechin, the 3′- and/or 4′ carbons are the probable positions of hydroxyl groups as well as sulfation, *O*-methylation and *O*-glucuronidation. Some of the radiolabeled HPLC peaks in urine from some, but not all, volunteers contained more than one metabolite that were resolved when detected by MS but not by the radioactivity monitor because the response of the detector was, of necessity, averaged over a ~10s period through the operation of a time constant [9].

As shown in Supplementary Table S2, the following 11 structurally related (−)-epicatechin metabolites (SREM) were identified at MSI MI level 1 via co-chromatography with a reference compound and matching MS^2 spectra: (−)-epicatechin-3′-*O*-β-D-glucuronide, (−)-epicatechin-7-*O*-β-D-glucuronide, 3′-*O*-methyl-(−)-epicatechin-5-*O*-β-D-glucuronide, 3′-*O*-methyl-(−)-epicatechin-7-*O*-β-D-glucuronide, (−)-epicatechin-3′-sulfate, (−)-epicatechin-5-sulfate, (−)-epicatechin-7-sulfate, 3′-*O*-methyl-(−)-epicatechin-5-sulfate, 3′-*O*-methyl-(−)-epicatechin-7-sulfate, 4′-*O*-methyl-(−)-epicatechin-5-sulfate and 4′-*O*-methyl-(−)-epicatechin-7-sulfate. Also identified in this manner, using reference compounds isolated from urine, were the 5-carbon-side chain ring fission metabolites (5C-RFM) 5-(4′-hydroxyphenyl)-γ-valerolactone-3′-sulfate, 5-(4′-hydroxyphenyl)-γ-valerolactone-3′-*O*-glucuronide, and 5-(3′-hydroxyphenyl)-γ-valerolactone-4′-*O*-glucuronide, 5-(phenyl)-γ-valerolactone-3′-sulfate and 5-(phenyl)-γ-valerolactone-3′-*O*-glucuronide. Partial identification at MSI MI level 2 was obtained for a further six 5C-RFMs namely two 5-(phenyl)-γ-valerolactone-sulfate-*O*-glucuronides, a 5-(hydroxyphenyl)-γ-hydroxyvaleric acid-*O*-glucuronide, two 5-(hydroxyphenyl)-γ-hydroxyvaleric acid-sulfates and a 5-(phenyl)-γ-hydroxyvaleric acid-sulfate. MSI MI level 1 identifications were also achieved with 3-(3′-hydroxyphenyl)-3-hydroxypropionic acid, hippuric acid and 3′-hydroxyhippuric acid whereas level 2 identification was obtained with a hydroxyphenylacetic acid-sulfate (Supplementary Table S2). Quantification of these compounds, or groups of compounds, was based on the size of the appropriate radiolabeled HPLC peak. The structures of metabolites identified at the MSI MI level 1 are presented in Supplementary Fig. S2.

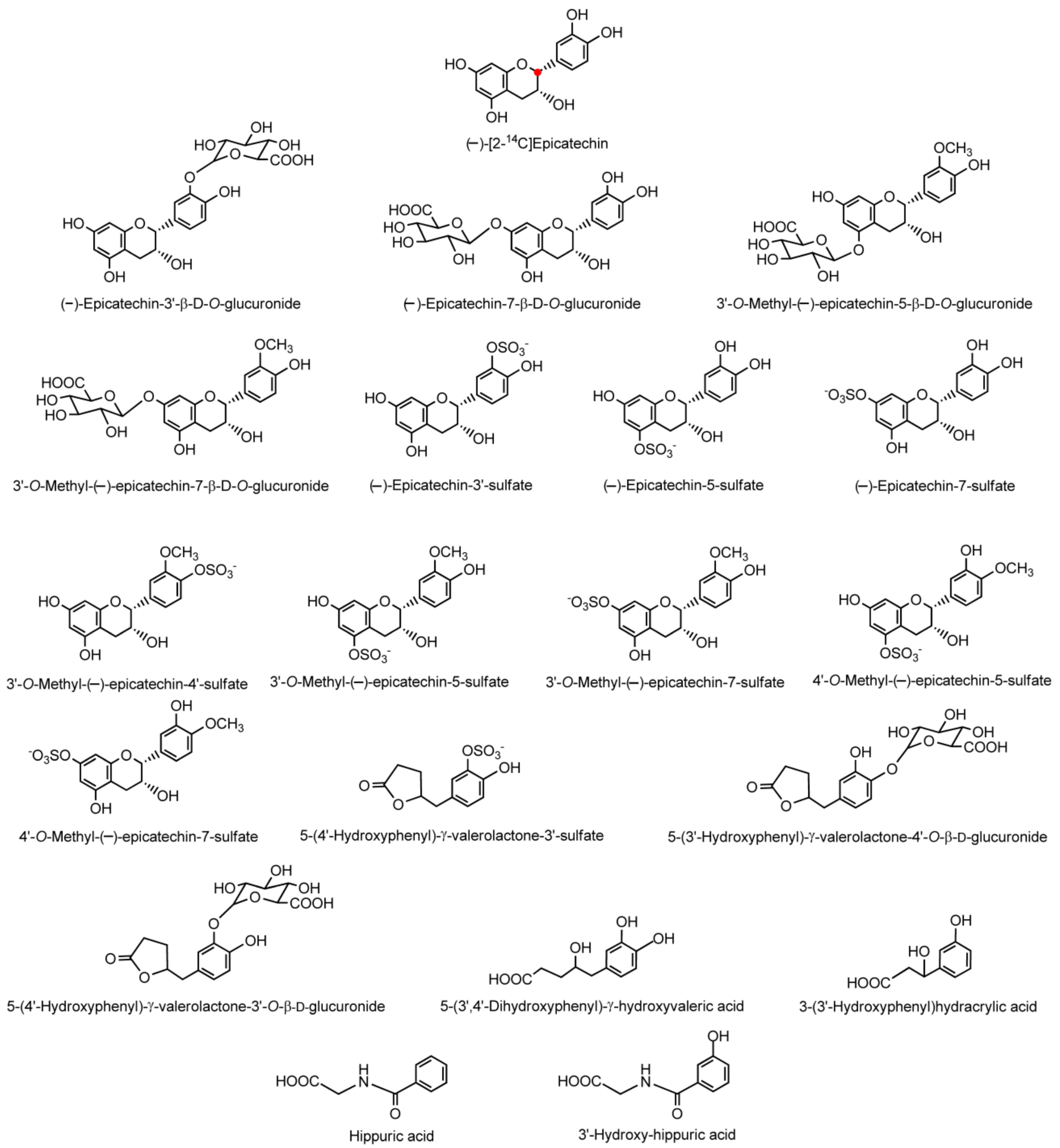

Supplementary Figure S2. Structures of [2-^{14}C](−)-epicatechin and its human metabolites.

HPLC-MS analysis of plasma

The level of radioactivity in plasma was low compared to that excreted in urine and, as a consequence, it was not a practical option to use an on-line radioactivity monitor with HPLC. Specific SREMs and 5C-RFMs were therefore analyzed by HPLC with MS in the selected reaction monitoring mode using a 150 mm x 4.6 mm i.d. 2.6 µm Kinetex Phenyl-Hexyl column (Phenomenex) eluted at a flow rate of 1 mL/min with a 30 min gradient of

5-20% acetonitrile in 0.1 % formic acid. Supplementary Table S2 provides details of HPLC retention times and the *m/z* values that were monitored.

Supplementary Table S2. Detection of (−)-epicatechin metabolites. HPLC retention times and selected reaction monitoring ions used for the HPLC-MS analysis of (−)-epicatechin metabolites in plasma.

**(−)-Epicatechin Metabolites**	**Rt (min)**	**$[M–H]^−$ *(m/z)***	**MS^2 ions *(m/z)***
Structurally related (−)-epicatechin metabolites (SREM)			
(−)-Epicatechin-3′-*O*- β-D-glucuronide	14.4	465	289, 245
(−)-Epicatechin-7-*O*- β-D-glucuronide	10.2	465	289, 245
(−)-Epicatechin-3′-sulfate	19.5	369	289, 245
(−)-Epicatechin-5-sulfate	16.4	369	289, 245
(−)-Epicatechin-7-sulfate	18.2	369	289, 245
3′-*O*-Methyl-(−)-epicatechin-4′-sulfate	19.9	383	303
3′-*O*-Methyl-(−)-epicatechin-5-sulfate	22.5	383	303
3′-*O*-Methyl-(−)-epicatechin-7-sulfate	23.9	383	303
4′-*O*-Methyl-(−)-epicatechin-5-sulfate	25.4	383	303
4′-*O*-Methyl-(−)-epicatechin-7-sulfate	26.9	383	303
3′-*O*-Methyl-(−)-epicatechin-5-*O*-β-D-glucuronide	16.0	479	303
3′-*O*-Methyl-(−)-epicatechin-7-*O*- β -D-glucuronide†	14.9	479	303
5-carbon side chain ring fission metabolites (5C-RFM)			
5-(4′-Hydroxyphenyl)- γ-valerolactone-3′-*O*-sulfate	20.1	287	207, 163
5-(3′-Hydroxyphenyl)- γ-valerolactone-4′-*O*- β-D-glucuronide	12.7	383	207, 163
5-(4′-Hydroxyphenyl)- γ-valerolactone-3′-*O*- β-D-glucuronide	14.5	383	207, 163
5-(Phenyl)- γ-valerolactone-sulfate-*O*- β-D-glucuronide-I	18.2	463	383, 287
5-(Phenyl)- γ-valerolactone-sulfate-*O*- β-D-glucuronide-II	21.0	463	383, 287
5-(3′-Hydroxyphenyl)- γ-hydroxyvaleric acid-4′-sulfate	10.8	305	225, 207
5-(4′-Hydroxyphenyl)- γ-hydroxyvaleric acid-3′-sulfate	11.2	305	225, 207
5-(3′-Hydroxyphenyl)-γ-hydroxyvaleric acid-4′-*O*- β-D-glucuronide	7.7	401	225

Supplementary References

1 Nadler, S. B., Hidalgo, J. H. & Bloch, T. Prediction of blood volume in normal human adults. *Surgery* **51**, 224-232 (1962).

2 Ottaviani, J. I., Momma, T. Y., Kuhnle, G. K., Keen, C. L. & Schroeter, H. Structurally related (-)-epicatechin metabolites in humans: assessment using de novo chemically synthesized authentic standards. *Free Radic Biol Med* **52**, 1403-1412, doi:10.1016/j.freeradbiomed.2011.12.010 (2012).

3 Borges, G. *et al.* Bioavailability of multiple components following acute ingestion of a polyphenol-rich juice drink. *Mol Nutr Food Res* **54 Suppl 2**, S268-277, doi:10.1002/mnfr.200900611 (2010).

4 Stalmach, A. *et al.* Absorption, metabolism, and excretion of green tea flavan-3-ols in humans with an ileostomy. *Mol Nutr Food Res* **54**, 323-334, doi:10.1002/mnfr.200900194 (2010).

5 Llorach, R. *et al.* Metabolomics study of human urinary metabolome modifications after intake of almond (Prunus dulcis (Mill.) D.A. Webb) skin polyphenols. *J Proteome Res* **9**, 5859-5867, doi:10.1021/pr100639v (2010).

6 Sang, S. & Yang, C. S. Structural identification of novel glucoside and glucuronide metabolites of (-)-epigallocatechin-3-gallate in mouse urine using liquid chromatography/electrospray ionization tandem mass spectrometry. *Rapid Commun Mass Spectrom* **22**, 3693-3699, doi:10.1002/rcm.3786 (2008).

7 van der Hooft, J. J. *et al.* Structural elucidation and quantification of phenolic conjugates present in human urine after tea intake. *Anal Chem* **84**, 7263-7271, doi:10.1021/ac3017339 (2012).

8 Sumner, L. W. *et al.* Proposed minimum reporting standards for chemical analysis Chemical Analysis Working Group (CAWG) Metabolomics Standards Initiative (MSI). *Metabolomics* **3**, 211-221, doi:10.1007/s11306-007-0082-2 (2007).

9 Reeve, D. R. & Crozier, A. Radioactivity monitor for high-performance liquid chromatography. *J. Chromatogr. A* **137**, 271-282, doi:doi:10.1016/S0021-9673(00)81350-3 (1977).
